# Real-Time Energy Efficient Hand Pose Estimation: A Case Study

**DOI:** 10.3390/s20102828

**Published:** 2020-05-16

**Authors:** Mhd Rashed Al Koutayni, Vladimir Rybalkin, Jameel Malik, Ahmed Elhayek, Christian Weis, Gerd Reis, Norbert Wehn, Didier Stricker

**Affiliations:** 1 Microelectronic Systems Design Research Group, Department of Electrical and Computer Engineering, Technische Universität Kaiserslautern, 67663 Kaiserslautern, Germany; rybalkin@eit.uni-kl.de (V.R.); weis@eit.uni-kl.de (C.W.); wehn@eit.uni-kl.de (N.W.); 2 German Research Center for Artificial Intelligence, DFKI, 67663 Kaiserslautern, Germany; jameel.malik@dfki.de (J.M.); ahmed.elhayek@dfki.de (A.E.); gerd.reis@dfki.de (G.R.); didier.stricker@dfki.de (D.S.); 3 Department of Informatics, Technische Universität Kaiserslautern, 67663 Kaiserslautern, Germany; 4 School of Electrical Engineering and Computer Science (SEECS), National University of Sciences and Technology (NUST), Islamabad 44000, Pakistan

**Keywords:** hardware architecture, FPGA, Zynq, UltraScale+, HLS, PyTorch, CNN, deep learning, hand pose estimation

## Abstract

The estimation of human hand pose has become the basis for many vital applications where the user depends mainly on the hand pose as a system input. Virtual reality (VR) headset, shadow dexterous hand and in-air signature verification are a few examples of applications that require to track the hand movements in real-time. The state-of-the-art 3D hand pose estimation methods are based on the Convolutional Neural Network (CNN). These methods are implemented on Graphics Processing Units (GPUs) mainly due to their extensive computational requirements. However, GPUs are not suitable for the practical application scenarios, where the low power consumption is crucial. Furthermore, the difficulty of embedding a bulky GPU into a small device prevents the portability of such applications on mobile devices. The goal of this work is to provide an energy efficient solution for an existing depth camera based hand pose estimation algorithm. First, we compress the deep neural network model by applying the dynamic quantization techniques on different layers to achieve maximum compression without compromising accuracy. Afterwards, we design a custom hardware architecture. For our device we selected the FPGA as a target platform because FPGAs provide high energy efficiency and can be integrated in portable devices. Our solution implemented on Xilinx UltraScale+ MPSoC FPGA is 4.2× faster and 577.3× more energy efficient than the original implementation of the hand pose estimation algorithm on NVIDIA GeForce GTX 1070.

## 1. Introduction

The markerless 3D hand pose estimation (i.e., the ability to track and estimate the position of a hand pose without using any special markers) is increasingly gaining importance for human machine interaction (HMI) nowadays [1,2,3], which has many interesting applications such as manipulating with virtual objects in virtual environments or handling with real objects using a robotic arm. In Computer-Aided Design (CAD), for instance, a recent method has tried to minimize the use of the mouse, thereby allowing the designers to move their hands freely in the air for drawing [4]. In the field of robotics, an intriguing application example is shadow dexterous hand where a robotic arm has to mimic the human hand poses accurately [5]. Hand pose estimation can be helpful for communication in sign language with deaf and mute people [6]. Accurate 3D hand pose tracking is also important for smart interactions using wearable devices such as Google Glass and VR headsets. In-air signature verification for VR application is another interesting domain where hand pose tracking plays an important role [7].

Recently, depth-based deep hand pose estimation methods have achieved the state-of-the-art accuracy on public benchmarks [8] due to the significant advancement in deep learning in the recent years and the availability of low-cost depth cameras. Typically, Convolutional Neural Network (CNN)-based hand pose estimation methods are implemented and executed on the CPUs and/or Graphics Processing Units (GPUs). However, these platforms are not feasible in practice because of their high computational costs, longer run-time and high power consumption. Moreover, it is difficult to embed the GPUs into tiny, portable platforms due to their large size and low energy efficiency. These drawbacks constrain the deployment of the GPU-based systems for the practical application scenarios in the industrial environments such as HMI and shadow dexterous hand.

In order to overcome these challenges, we choose the FPGA as an underlying hardware platform for the following reasons and we efficiently map the hand pose estimation CNN to it. First of all, in contrast to the CPU-based context switching and multi-threading, the physical parallel execution as well as pipelining in the FPGA make it the best choice for the real-time processing applications as compared to GPUs and CPUs. Furthermore, many practical applications, as mentioned before, require the deployment of hand pose estimation systems on portable platforms with limited hardware resources and power budget. Lastly, the deployment of FPGA, in contrast to GPUs, does not require a bulky PC or server. Thus, FPGA is considered more suitable for portability in terms of size as well. Although the FPGAs are less energy efficient as compared to their ASIC counterparts, ASICs are considered more costly and less flexible for after-development updates [9]. In this work, we address several challenges during the hardware implementation, such as minimizing the area occupied on chip which reflects on power consumption, as well as minimizing the latency of hand pose recognition. We provide the first FPGA-based 2D CNN accelerator for hand pose estimation which predicts the 3D coordinates of hand joints from a single depth image.

To develop such an FPGA implementation, we compress the 2D CNN by applying dynamic quantization techniques. Instead of fine-tuning an already trained network, this step involves retraining the CNN from scratch with constrained bitwidths for the weights and activations. This process is called quantization-aware training (QAT). The resulting compressed CNN is much smaller than the full-precision CNN while the accuracy is only slightly decreased. Once the network is compressed, we design an efficient streaming hardware architecture. This step involves an iterative process of exploring the design space, seeking to find the best hardware-aware representation for the different CNN layers. In order to speed-up the design process, we use the High Level Synthesis (HLS) to design the hardware architecture for the chosen CNN. We deploy the CNN hardware model on Xilinx UltraScale+ MPSoC FPGA. Consequently, we synthesize and integrate the overall system, and we design the needed software that runs on the processing system (PS) for preprocessing and input/output interfacing with the programmable logic (PL).

The following points summarize the contribution of this paper:

- A compressed, fixed-point version of the hand pose estimation CNN network that is 5.3× smaller as compared with the uncompressed, floating point CNN. This squeezed version of the GPU-based CNN adopts customized bitwidths for weights and activations, and requires the minimum amount of computation in the convolutional layer i.e., depthwise separable convolution.
- A hardware architecture that is 4.2× faster and 577.3× more energy efficient than the originally proposed CNN implementation of hand pose estimation algorithm on NVIDIA GeForce GTX1070. This architecture exploits the parallelization in FPGA in order to speedup the inference of hand joints coordinates based on Zynq UltraScale+ XCZU9EG MPSoC using High-Level Synthesis (HLS).

Since this is the first FPGA-based hand pose estimation implementation, our work contributes to the field of embedded vision by showing how fast and energy efficient an embedded FPGA implementation can be as compared to the conventional GPU implementations and other embedded platforms.

## 2. Related Work

To the best of our knowledge, we are not aware of any implementation of CNN-based hand pose estimation on FPGA.

**Hand Pose Estimation:** Our implementation of hand pose estimation CNN on FPGA is originally based on the work of Malik et al. [10] and inspired by it. In this work, Malik et al. have shown enhanced precision over the state-of-the-art using a unified dataset. In fact, Oberweger et al. [11] tried different CNN topologies for the 3D hand pose estimation, and the basic CNN in [10] is similar to one of the CNNs in [11], in which only NYU and ICVL datasets were used to train and test the CNN.

**Network Quantization:** Krishnamoorthi [12] discussed different quantization techniques i.e., quantization-aware training and fine tuning (post quantization). He showed that quantization-aware training can provide higher accuracy than post quantization training schemes. In [13], Hubara et al. introduced a training method for the quantized neural networks. Furthermore, they have illustrated different classification performance for different network topologies such as AlexNet and GoogleNet.

**CNN Implementation on FPGA:** Venieris et al. showed in their survey [14] that hardware architecture for CNN implementation on FPGA can be categorized into two main categories: streaming architectures and single computation engine. In streaming architecture [15,16,17,18,19,20,21,22], each layer is mapped to a hardware block and these hardware blocks are connected with each others via stream pipes. In fact, our architecture in this work is a simplified version of the FINN architecture [22].

## 3. Overview of The Proposed Approach

In this section, we provide an overview of the CNN-based hand pose estimation algorithm and its implementation on the FPGA.

### 3.1. CNN-Based Hand Pose Estimation Algorithm

**Figure 1:**
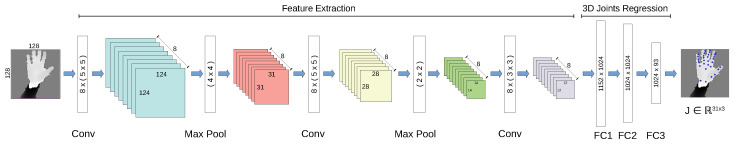
The architecture of Convolutional Neural Network (CNN)-based hand pose estimation algorithm. The CNN takes a 128 × 128 input preprocessed image. It consists of 3 convolution layers each followed by an ReLU activation and max pooling. Afterwards, there are 2 fully connected layers with ReLU activation. A third fully connected layer (joint regression layer) regresses 3D joint positions. Conv stands for a convolution layer. Each conv is followed by a ReLU activation. FC denotes a fully connected layer.

In this subsection, we explain the CNN architecture [11] which we adopt for the FPGA-based hardware implementation. Figure 1 illustrates the complete topology of the mentioned CNN. The input to this CNN is a preprocessed depth image of size 128 × 128. This CNN consists of 3 convolution layers using 5 × 5, 5 × 5, 3 × 3 kernels, respectively. ReLU activation is applied to the output of each convolution layer. The first two convolution-ReLU layers are connected to max pooling layers with filter size 4 × 4, 2 × 2, respectively. It should be noted that depthwise convolution [23] is used in the convolution layers. In other words, each input feature map is processed using one kernel independently, and no mutual operation across different feature maps is performed. We will provide more details about our implementation of depthwise convolution in Section 5.1.1. The 8 output feature maps are then flattened into a 1D vector which represents the input of the fully connected layer set. This set consists of 3 fully connected layers of size 1152, 1024 and 93, respectively. The first two layers are attached to the ReLU activation layers.

However, the last fully connected layer (joint regression layer) is responsible for generating the 93 joint coordinates (i.e., 31 3D joint positions) and needs therefore no activation function.

### 3.2. Design Process Overview

**Figure 2:**
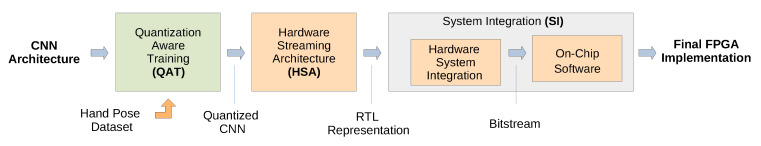
Design process overview; the first box illustrates the software-level design phase, while the other boxes illustrate the hardware related design phase. The first stage is quantization-aware training (QAT) in which we decrease the CNN memory demand as well as the computation time on the hardware. The second stage is hardware streaming architecture (HSA) where the underlying hardware structure is designed for the CNN. In system integration (SI) stage, the programmable logic PL and the processing system PS are brought together and the interface with the memory is configured through the hardware system integration sub-stage. Furthermore, the on-Chip Software is developed for preprocessing and interfacing with the peripherals.

Figure 2 shows an overview of the hardware design process for implementing the aforementioned CNN on the FPGA. The design process consists mainly of three major design stages. The first stage is quantization-aware training (QAT). This stage requires the CNN layer description as well as the hand pose dataset. In this stage, we quantize the CNN in order to decrease its size. This is done by re-training the CNN from scratch under the constraint of limited weights and activations bitwidth(s). As a result, we acquire a lightweight trained version of the CNN with fixed-precision weights and activations. The second stage is the core stage in which we design the hardware streaming architecture (HSA) and test it by simulation. We employ high level synthesis (HLS) which provides the register transfer level (RTL) representation of the CNN. In order to design the hardware module that replicates the quantized CNN behavior, the CNN topology and the quantized parameters are needed. As a result, the RTL hardware module is generated. Subsequently, the third stage is the SI which stands for system integration. This stage consists of two sub-stages. In the first sub-stage of the SI (hardware system integration) we integrate the system. In other words, we bring together the different System-on-Chip (SoC) components such as the programmable logic (PL) and the processing system (PS). Furthermore, we specify and configure the interface protocol between the aforementioned components and the off-chip DRAM memory. The outcome of this stage is the bitstream needed to configure the FPGA, along with the hardware description. Although the interface between PS and PL is configured, the software that runs on the PS is still missing. This is where the second sub-stage (on-chip software) comes into play. In this stage, we develop the preprocessing and interface handling script for the PS. Consequently, the final FPGA implementation is ready for on-board deployment.

## 4. Software Design Process

In this section, we first explain the training and testing process for the full-precision CNN. Afterwards, we illustrate the quantization-aware training process and the quantization function.

### 4.1. Full-Precision CNN Training and Testing

Before the quantization-aware training process, it is important to obtain a full-precision version of the CNN within the accepted error limit. The full-precision CNN is considered a baseline for comparison with other customized compressed CNN models. In this work, we use PyTorch [24] to build, train and test the CNN. The hyper parameters for the CNN were chosen as reported in [25].

#### 4.1.1. Network Training

Table 1 shows the chosen training hyper parameters.

We train the hand pose estimation CNN on NVIDIA GeForce GTX 1070 GPU with 12 GB. Stochastic Gradient Descent (SGD) is used to feed the network forward with batch size of 256 samples every time before the back propagation takes place. A learning rate of 0.005 is chosen for gradient calculation. In order to speed up the learning, SGD uses momentum equals to 0.9 which is normally a recommended value. The error criterion used here is mean square error (MSE). We use the NYU hand pose dataset [26] to train and test the CNN. This dataset offers RGBD images along with their ground-truth 3D joint coordinates. The training set contains 72,757 frames, while the test set contains 8252 frames. The ground-truth values correspond to the *x*,*y* and *z* coordinates for each of the 31 joints of the hand, thus 93 values for each frame.

#### 4.1.2. Network Testing

In the testing phase, each image in the test set is fed in to the CNN, and the corresponding estimated coordinates of the 31 3D joints are calculated. Afterwards, the performance of the network is assessed by calculating the average single joint Euclidean error over all testset frames, measured in mm. The average joint error for direct joint regression turns out to be 17.2 mm, which is exactly the same error calculated by Zhou et al. in [25]. Further details about run-time delay and power consumption will be presented later in Section 6.

### 4.2. Quantization-Aware Training (QAT)

The trained CNN model obtained so far is a full-precision model that employs floating point (float) data type. In other words, 32 bits are used for representing each value in the model. In this case, the total model size can be calculated as the total number of parameters times 32 bits. The more parameters the model has, the larger will be the size, which makes it difficult to deploy CNNs on portable platforms even for small CNNs. Therefore, we need to find a way to decrease the size of the model with as little impact on accuracy as possible, and here comes the role of quantization. Although the full-precision provides the best accuracy in most cases, there are numerous drawbacks of using the full-precision CNNs. First of all, the more bits are used to represent the values, the more memory space is needed to store them. Secondly, when deploying this CNN on a portable device, the power consumption might be higher due to the data transfer as well as the arithmetic operations, which enormously reflects on the battery usage. Finally, the floating point numbers need a special handling process when added, compared or multiplied, which may result in additional unneeded delays. All these reasons urge the need of having a compact trained CNN model in which a negligible amount of accuracy is sacrificed for a gain in space, power and execution time. For this purpose, a quantization based training is to be performed.

Quantizing a neural network is the process of representing the CNN parameters with a fewer number of bits with only a slight or negligible degradation in the performance, especially for important applications as mentioned earlier in the introduction. Instead of using the floating point arithmetic in the quantized CNN, the fixed point representation of the different weights, biases and activation functions is applied. A fixed point value is represented as N=[I.F], where (*I*) is the integer part, (*F*) is the fractional part and (.) is the decimal point. Therefore, the total number of bits used to represent the fixed point value i.e., the word length ($L_{N}$) is given as the sum of the number of integer bits ($L_{I}$) and fractional bits ($L_{F}$):

(1) $$L_{N}=L_{I}+L_{F}$$

In order to decide a suitable bitwidth for quantization, we go through an iterative design space exploration with different widths for various coefficients. A trade-off between the number of bits and the CNN inference accuracy constrains the choices of quantization bitwidths. In principle, not all the layers in the model have to be quantized using the same bitwidth. For example, the bitwidth for the fully connected layer parameters can be different from that of convolution layer kernels. This is called “Dynamic Quantization”, in contrast to “Static Quantization” where all the layers use the same quantization bitwidth.

### 4.3. Linear Quantization Function

The unsigned linear quantization function [27] converts a full-precision positive value *x* to an $L_{x}$ bit fixed point value $\hat{x}$ by binary-shifting, rounding and cropping as follows:

(2) $$\hat{x}=quant\left(x,L_{x}\right)=clamp\left(\frac{round(x\times 2^{L_{F}})}{2^{L_{F}}},V_{L},V_{H}\right)$$

The rounding function round() calculates the nearest integer to a given floating point value, while $L_{x}$ and $L_{F}$ are the desired fixed point representation parameters of $\hat{x}$. First of all, *x* is multiplied by $2^{L_{F}}$ (implemented as left-shift by $L_{F}=L_{x}-L_{I}$ bits) in order to allocate all the needed bits on the left side of the decimal point temporarily. Then, we throw away the extra fractional bits beyond $L_{F}$ bits by rounding. Afterwards, the result is divided by $2^{L_{F}}$ (implemented as right-shift by $L_{F}$ bits) in order to get the correct quantized value. Finally, the result is cropped using clamp() function in order to get rid of the extra integer bits beyond the $L_{I}$ bits (if any). The clamp() function clips the input between a minimum value $V_{L}$ and a maximum value $V_{H}$ as follows:

(3) $$clamp\left(x,V_{L},V_{H}\right)=\left\{\begin{array}{cc} V_{L} & x<V_{L}\\ x & V_{L}\leq x\leq V_{H}\\ V_{H} & x>V_{H} \end{array}\right.$$

The clamping limits $V_{L}$ and $V_{H}$ for the fixed point quantization function are the minimum and maximum representable values using $L_{I}$ integer bits:

(4) $$\begin{array}{c} V_{L}=0\\ V_{H}=2^{L_{I}}-2^{-L_{F}} \end{array}$$

In case of the negative value quantization, the most significant bit (MSB) is reserved as sign bit and the signed quantization function has to be used. This can be obtained by correcting Equation (1) to be $L_{x}=L_{I}+L_{F}+1$ and the lower cropping limit in Equation (4) to be $V_{L}=-2^{L_{I}}$.

#### Quantization-Aware Training Process

The quantization-aware training process is similar to the full-precision training process and uses the same dataset. However, the main difference is that the quantization method mentioned in [13] is to be used. Based on the quantization function, the input image is quantized, and so are the convolution kernels, biases and weights of the fully connected layer. The training starts using a full-precision version of the CNN. A copy of this full-precision CNN is kept for later use. The CNN is then quantized and used for forward propagation of a batch of quantized input images. Next, the mean square error (MSE) loss function is calculated for the back propagation. In this step, the gradients of the loss function are calculated with respect to the quantized weights as they were used for the forward propagation. The last step is the weight update which takes place on the full-precision parameters rather than on their quantized version. After the completion of the software implementation and the quantization, we highlight the hardware aspect mentioning the FPGA implementation process and the encountered challenges.

## 5. Hardware Design Process

In this section, we explain the design of the hardware streaming architecture for the hand pose estimation CNN and the hardware architecture for different CNN layers. We also highlight the inter-layer packing technique that improves the overall throughput in the network. Furthermore, we explain how the activations were quantized. We also provide some details regarding the hardware platform selection and the system integration.

### 5.1. Hardware Streaming Architecture Design (HSA)

**Figure 3:**
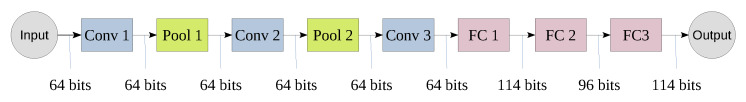
Streaming Architecture. Each CNN layer is mapped into a hardware block, and the hardware blocks are connected to each others via stream channels. The bitwidth of each stream is shown on this figure.

In the streaming architecture, each layer of the CNN is mapped to a separate hardware block and the blocks are connected to each other via stream channels to form a pipeline (as shown in Figure 3). Furthermore, the implementation of each layer can be optimized independently and the data can be streamed between layers on the fly. Particularly, a partial result of a layer can be directly streamed to the next layer without having to wait until the complete calculation is done [14]. As a result, this architecture facilitates the parallelism between layer blocks through pipelining.

#### 5.1.1. Convolutional and Pooling Layer Architecture

The convolutional layer as well as the pooling layer expect one or more feature maps as input and generate a number of output feature maps. Each feature map is scanned line by line, and each line is transferred to the next layer. This imposes that the first convolution or pooling operation cannot be completed until the appropriate number of lines are received by the CNN layer, which highlights the need to temporarily buffer the input lines. In case of convolutional layer, the kernel stride is less than its dimension. Therefore, the same input columns and/or rows are expected to be used by a later convolution operation using the same kernel. Instead of having to fetch the same set of input values from the previous feature maps repeatedly, local buffering saves the additional time and space needed by the previous layer. Therefore, a line buffer and a window buffer are needed. The line buffer is a special memory responsible for storing specific lines from previous feature maps locally. The dimensions of a line buffer depend on the kernel size as well as the size of the input feature map. The number of lines in a line buffer should be equal to the height of the kernel so that enough values can be covered by the kernel vertically. Furthermore, the number of columns in the line buffer should be the same number of columns in the input feature map(s). This is because a whole line should be stored before storing the next line [28,29]. To support parallelization, an array of line buffers, where each line buffer corresponds to an input feature map, is used. This could be thought of as a 3 dimensional line buffer. While the data is being buffered by the line buffer, the calculation of the concerned layer should take place. The window buffer is a small memory that has the same dimensions as the convolution or pooling kernel. This memory is responsible for preparing the data needed by the kernel to perform the convolution or the pooling.

The window buffer follows the same shifting pattern used by the kernel over the image. In fact, the window buffer copies the pixels required by the operation from the line buffer. These pixels are processed by the kernel, and the result is finally ready to be transferred to the next layer. Due to the hardware level parallelization in FPGA, 8 window buffers can sample the 8 line buffers that correspond to the 8 input channels, and 8 kernels can be applied on these window buffers in parallel. Resulting in 8 output values per clock cycle.

**Figure 4:**
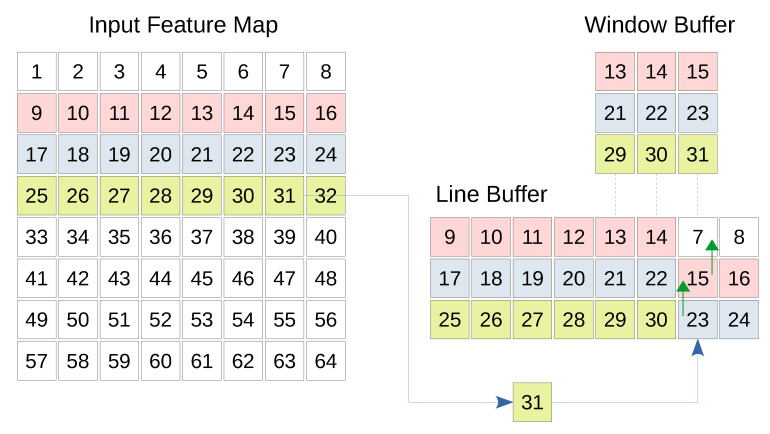
Line buffer and window buffer architecture. This figure illustrates an example of a single line buffer and a single window buffer for a single input feature map. In this example, the 4th line of the input feature map is being streamed value by value to the line buffer. Specifically, the input value 31 is streamed from the input feature map to the appropriate location in the line buffer. The old values (23 and 15) in the line buffer are shifted up to the appropriate places in their original lines, while the old value 7 is no more needed. The window buffer copies the set of line buffer values that correspond to the current operation (convolution or pooling).

Figure 4 shows the basic structure of the line and window buffer where the fourth line of the input map is being buffered. Furthermore, the window buffer is highlighting a 3 × 3 region which will be treated by a kernel of the same size. Whenever a new input comes, the corresponding column is shifted up allowing the pixel to be inserted.

The idle time of a particular convolution or max pooling layer is the total number of input values that must be streamed to the line buffer in order for the convolution or max pooling computation engine to start or continue working. For example, in case of 3×3 convolution, the operation cannot be started until the first two lines and the first three values of the third line are already received by the line buffer. Furthermore, the computation engine is paused at the end of the third line because it has to wait for the first three values of the fourth line to be available. The same applies for the rest of the lines. This imposes additional waiting delays during which the computation engine is idle. In fact, every time the layer operation is executed, an output value is generated. Thus, we can consider the size of the output feature map as the number of times the layer operation was executed. This way, we can calculate idle time $\Delta_{idle}$ of the layer as the difference between the total buffering time $T_{total}$ calculated as the size of the input feature map, and the effective computation time $T_{eff}$ given as the output feature map size, as shown in Equation (5).

(5) $$\begin{array}{cc} \Delta_{idle} & =T_{total}-T_{eff}=C_{in}\times H_{in}\times W_{in}-H_{out}\times W_{out}\\ \; & =C_{in}\times H_{in}\times W_{in}-\frac{H_{in}-K_{H}-\left(K_{H}-1\right)\times \left(d-1\right)}{s}\times \frac{W_{in}-K_{W}-\left(K_{W}-1\right)\times \left(d-1\right)}{s} \end{array}$$

where $C_{in}$, $H_{in}$ and $W_{in}$ are the number of input channels, the input height and the input width, respectively, $H_{out}$ and $W_{out}$ are the output height and width, respectively, $K_{H}$ and $K_{W}$ are the kernel height and width, respectively and finally *s* and *d* are the stride and dilation, respectively. It is noted that the idle time depends on the number of the input feature maps but irrelevant to the number of the output feature maps. The previous equation can be further simplified to Equation (6) by assuming that the input feature maps and the kernels are all squares ($W_{in}=H_{in}$ and $K_{W}=K_{H}$), which is our case (see Figure 1).

(6) $$\begin{array}{cc} \Delta_{idle} & =C_{in}\times H_{in}^{2}-\frac{{\left(H_{in}-d\times \left(K_{H}-1\right)-1\right)}^{2}}{s^{2}} \end{array}$$

The physical time interval equivalent to $\Delta_{idle}$ can be calculated as $\Delta_{idle}\times No.Clk\times ClockPeriod$, where No.Clk is the number of clock cycles needed to transfer one input pixel to the line buffer. In the following paragraphs, we provide architectural details on how the stride and group convolution are handled and how they affect the idle time in a particular layer. Furthermore, we provide architectural information about the dilation and the skip/merge connections in Section 7.

• Stride

In case of the convolutional layers, the kernel is shifted by one pixel after each convolution operation horizontally or vertically (i.e., stride = 1). While in the first and the second max pooling layers, the stride is equal to the pooling filter sizes (i.e., stride = 4 and 2, respectively). In order to overcome the additional complexity imposed by the striding, we design a generic buffering engine that can be dynamically customized for different stride values. For this purpose, a row counter and a column counter are used to keep track of the row and the column to which the input value in the input feature map belongs. As the input values are streamed to the line buffer, the window buffer copies the chunk of values that are needed for the layer operation (i.e., convolution or max pooling) as illustrated in Figure 4. However, the decision to start the layer operation is based on the values of the row and the column counters. Specifically, the operation is allowed to be performed only when the row and column counters are multiples of the vertical and horizontal strides respectively. Algorithm A1 in Appendix A.1 shows how the stride operation is handled. In order to study the effect of the stride on the layer’s idle time, we differentiate the idle time with respect to the stride *s* in Equation (6):

(7) $$\begin{array}{cc} \frac{\partial \Delta_{idle}}{\partial s} & =\frac{2\times {\left(H_{in}-d\times \left(K_{H}-1\right)-1\right)}^{2}}{s^{3}}\geq 0 \end{array}$$

This means that the higher the stride, the longer the waiting time within the layer.

• Group Convolution

In group convolution, the input feature maps are arranged in groups, where each group is convoluted with its corresponding set of kernels. This way, each output feature map will be inferred from the input feature maps within the corresponding input group instead of being related to all input feature maps as in the standard convolution. No architectural modification for the line buffers or the window buffers is required, since the input still has to be buffered in the line buffers and then prepared in the window buffers for convolution. We introduce the “group router”, an array of demultiplexers that dynamically relate each window buffer to the correlative set of kernels. The routing decision inside the group router is based on the input group order as well as the input feature map order. In case of the first convolutional layer in our work, which produces 8 output channels from a single input channel, 8 convolutional kernels are used for the single input group. While in the second and the third convolutional layers, the standard convolution requires 64 kernels to produce 8 output channels. As mentioned in Section 3.1, depthwise convolution is used to reduce the the number of kernels from 64 to 8, where each kernel is convoluted with a single input feature map. The group convolution has no effect on the idle time $\Delta_{idle}$ of the layer since it does not effect the buffering scheme in the line buffer. Further details about the group convolution are available in Appendix A.3.

#### 5.1.2. Zero Padding Layer Architecture

**Figure 5:**
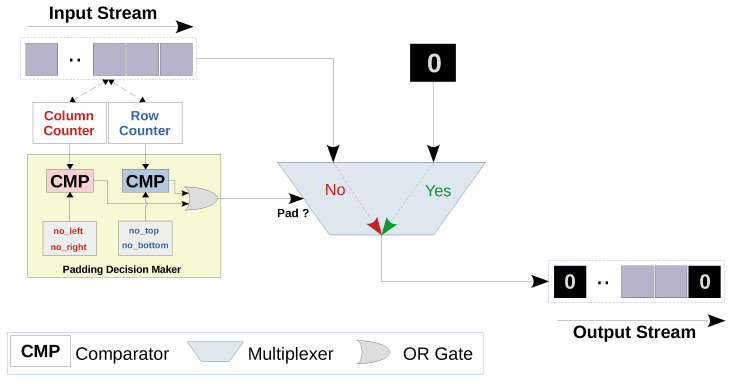
Zero padding layer streaming architecture. In this architecture, the input values are streamed in, and the padding decision maker controls the multiplexer based on the current row and column indices. If the padding should be performed, a zero value is streamed out and the input stream stalls. Otherwise, the input value is directly pushed as is to the output stream.

Zero padding is the process of increasing the size of a feature map by adding extra rows and/or columns of zeros at its borders. Furthermore, the dimensions of the input feature maps of a max-pooling layer should be multiples of the pooling filter dimensions, given that the strides are equal to the filter dimensions. Otherwise, the filter would not fit at some or all feature maps borders. In our work, we use a zero padding layer to pad the top and left borders in the input feature maps of the second pooling layer, as it uses a pooling filter of size 2 × 2 for the input dimensions of size 27 × 27. Figure 5 illustrates a simplified representation of the padding layer architecture. The multiplexer pushes a zero to the output stream when padding is needed, while it relays the input to the output otherwise. This decision is based on the values of two counters (namely, row and column counters). These counters keep track of the horizontal and vertical locations of each input value relatively to the corresponding output feature map. Algorithm A2 in Appendix A.2 shows a code snippet for zero padding.

#### 5.1.3. Fully Connected Layer Architecture

**Figure 6:**
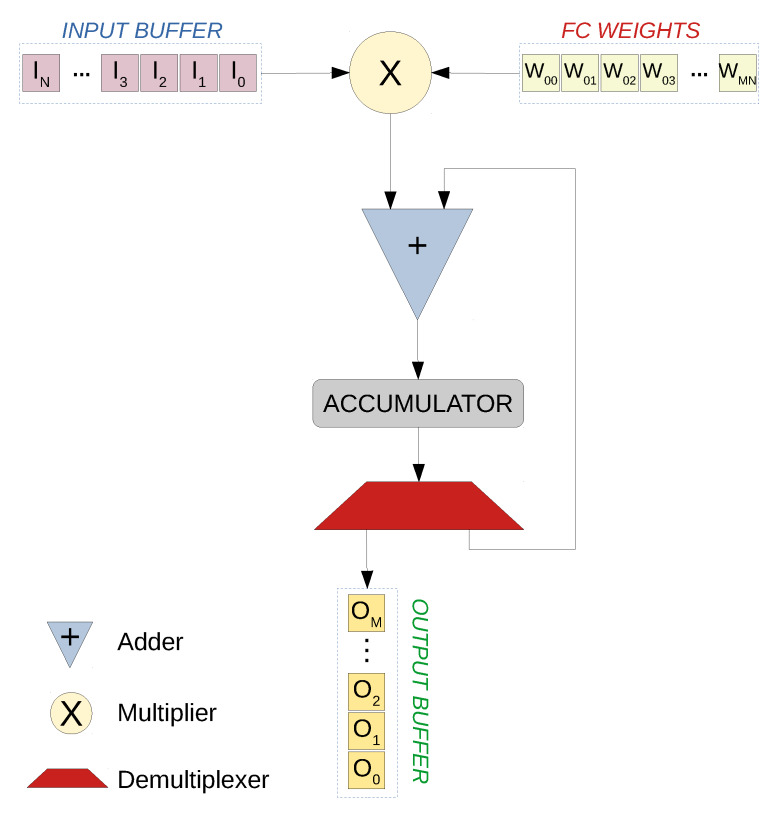
Fully connected layer streaming architecture. In this architecture, the input values are streamed in, and the corresponding weights are fetched from the local BRAM memories for the MAC operation that takes place in the multiplier and the adder. The result is accumulated with the help of the accumulator and the demultiplexer. Once the MAC operations are done, the demultiplexer pushes the result value to the output streaming buffer.

The core operation of a fully connected layer is multiply and accumulate operation, where each value in the input buffer is multiplied by the corresponding weights, and the results are then added to the accumulators and stored there. Each value is multiplied by a number of weights equal to the number of neurons in this layer. The basic architecture of this layer consists of a multiplier, an adder and an accumulator, as shown in Figure 6. When the multiplications are complete, the result value is passed to the ReLU activation function and then streamed out to the next layer via the output buffer. In some scenarios, such as in the last fully connected layer, no activation function is needed. To support parallelization, this architecture is instantiated a number of times equal to the parallelization degree. Multiplications will then occur in parallel and a number of accumulators are updated simultaneously.

#### 5.1.4. Inter-Layer Packing

Once an output value of a layer is ready, it is streamed to the next layer for processing. Nevertheless, this limits the throughput between layers to one result value per clock cycle at most. In order to increase the throughput across the CNN (and consequently decrease the latency), we apply the pack/unpack technique. In this technique, *N* output values of a layer ($L_{i}$) are concatenated together to form a single wider word, which is streamed within one clock cycle to the next layer (i+1). Consequently, the next layer (i+1) unpacks the concatenated word into its N values and performs the layer operation (e.g., convolution) in parallel. Theoretically, the inter-layer pipes have no bandwidth limit and any amount of data could be streamed from a layer to another. However, this is practically limited by the maximum achievable number of outputs at each clock cycle due to the limited hardware resources. As mentioned before, the first convolutional layer generates 8 output channels from a single input channel using 8 different convolutional kernels. If these 8 convolutions are done in parallel, the 8 output values can be concurrently streamed to the next layer within the same clock cycle. Along the rest of the CNN, the number of channels is fixed to 8 (Figure 1). Therefore, we choose N=8 so that the 8 output values can be packed together and transferred to the next layer, which in turn unpacks, performs layer operation and packs 8 output values. Each of these 8 values belong to a specific, single output channel of a layer i.e., to a single input channel of the next layer.

#### 5.1.5. Activation Quantization

The output of the convolutional layers and the fully connected layers is normally obtained by applying an activation function on the result of the multiply and accumulate operations in each layer. For the representation of an activation output in a particular layer, the same amount of bits used for the weights in that layer might not necessarily be enough. Therefore, the sufficient number of bits ($L_{N}=L_{I}+L_{F}$) has to be determined for the input as well as the output of each activation layer:

1. Convolutional Layer 1 and Pooling Layer 1 data type.
2. Convolutional Layer 2 and Pooling Layer 2 data type.
3. Convolutional Layer 3 data type.
4. Fully connected Layer 1 data type.
5. Fully connected Layer 2 data type.
6. Fully connected Layer 3 data type.

In order to determine the integer part length ($L_{I}$) for the activation quantization, profiling each layer’s output range for the whole testset should be performed. This is achieved by using a profiling function which observes each calculation in every layer and keeps a copy of the maximum and minimum result. For instance, for each kernel convolution in the first convolutional layer, the maximum and minimum results of the multiplication and addition operation are saved. This applies for each input test image as well as other layers. Afterwards, the range needed for each layer (*i*) in the worst case is calculated using Equation (8).

(8) $$Range_{i}=max_{i}-min_{i}$$

where $max_{i}$ and $min_{i}$ are the maximum and minimum output values of the layer (*i*), respectively. Eventually, the number of bits needed for the integer part is determined by applying Equation (9).

(9) $$L_{I}=\log_{2}\left(Range_{i}\right)$$

Now that ($L_{I}$) is known, we need to determine ($L_{F}$). For this purpose, we follow an iterative process for bitwidth exploration. It should be noted that we follow two different approaches for parameter quantization and activation quantization. In case of input and parameter quantization, we only constrain the input, weights and biases ($L_{N}$ already constrained) to certain bitwidths during training (QAT) and we use the quantization function mentioned in Equation (2). While for the activations, we explore the needed bitwidth $L_{N}$ by finding the needed $L_{I}$ and $L_{F}$ as mentioned.

### 5.2. System Integration (SI)

In this subsection, we provide a short description of the system integration process, including the platform selection, hardware system integration and on-Chip Software.

#### 5.2.1. Platform Selection

**Figure 7:**
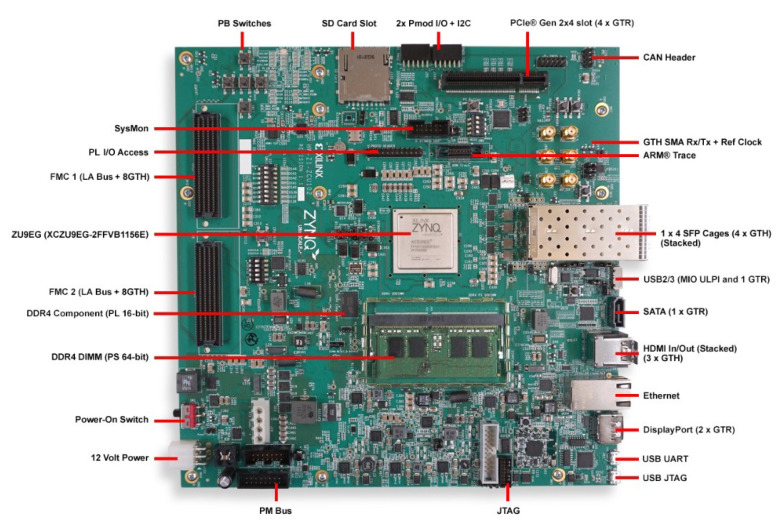
ZCU102 evaluation board [31].

Based on the CNN size estimation and the selection guide provided by Xilinx [30], ZCU102 Evaluation Board was chosen for the implementation of this project. Zynq^®^ UltraScale+™ XCZU9EG-2FFVB1156E MPSoC (multiprocessor System-on-Chip) is the core of this general purpose platform. In this platform, the PS is a multiprocessor System-on-Chip consisting of an ARM^®^ flagship Cortex^®^-A53 64-bit quad-core processor and a Cortex-R5 dual-core real-time processor along with high speed DDR4 SODIMM and component memory interfaces. The PL is basically the Field Programmable Gate Array (FPGA) which features 912 (Block RAM) BRAMs, 2520 DSP48, 274k (Look-Up Tables) LUTs and 548k (Flip-Flops) kFFs. Figure 7 shows an overview of the board and its components.

#### 5.2.2. Hardware System Integration and On-Chip Software

**Figure 8:**
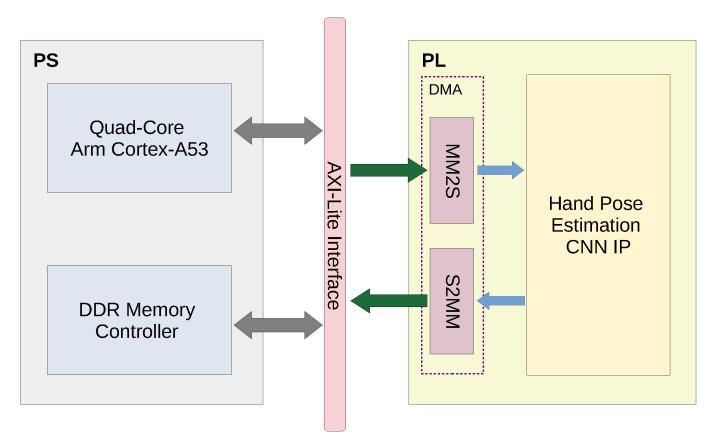
Hardware system integration; AXI-Lite interface provides the interconnection between the PS and the PL. DMA module is integrated in the PL. This module is responsible for converting the memory mapped input to AXI stream CNN input, as well as converting the AXI stream CNN output to a memory mapped output.

Figure 8 shows the architecture of the integrated system on chip. AXI-Lite interface provides the interconnection between the PS and the PL. The DMA module is integrated in the PL. This module is responsible for converting the memory mapped input to AXI stream CNN input, as well as converting the AXI stream CNN output to a memory mapped output. The (PS) is responsible for transferring the input image to the (PL) through AXI4 High Performance Interface. The software program is a C++ program that runs on the (PS), providing an interface to DRAM for writing images and reading inference results.

## 6. Results

In this section, we illustrate the results that we obtained along the design process depicted in Figure 2. Similarly to the design process, the results are split into two groups: software-related and hardware-related experiments and results.

### 6.1. Software-Related Experimental Setup and Results

In the following, we provide the training and testing results for the quantization process of the chosen CNN.

#### Training and Testing the Quantized CNN

Instead of trying different bitwidths randomly, we started with 16 bits as an initial value for the quantization. As long as the calculated error is within the accepted range, a fewer number of bit is chosen. Otherwise, training is run for more epochs until no more progress is observed. During the quantization trials, different bitwidths for different layers were also used. After going through this exploratory process, we found that the CNN that employs 12 bits for the quantization of weights and biases of the convolutional layers, 6 bits for the weights and biases of the fully connected layers and 8 bits for the input is the most suitable one. After 1000 epochs, the evaluation error turns out to be 17.75 mm. This implies that the quantized CNN is only 3% less accurate than its original full-precision ancestor. As a result, Table 2 illustrates a comparison between the total size needed by the original full-precision CNN and the quantized CNN.

### 6.2. Hardware-Related Experimental Setup and Results

In this subsection, we provide the run-time delay and energy efficiency analysis of the end-to-end hand pose estimation application after deployment on FPGA as compared to its GPU-based counterpart. For the GPU implementations, we use the NVIDIA GeForce GTX 1070. Furthermore, we illustrate the run-time and energy measurements for different GPU implementations which correspond to different batch sizes, and we highlight the most interesting cases among all the provided experiments. Additionally, we demonstrate the run-time delay and energy efficiency analysis as compared with Raspberry Pi 3B+ and NVIDIA Jetson Xavier as embedded platforms. Finally, we summarize the resource utilization analysis on FPGA.

#### 6.2.1. Comparison with NVIDIA GeForce GTX 1070 GPU

Since the original hand pose estimation CNN is trained and deployed on the powerful NVIDIA GeForce GTX 1070 GPU, it is beneficial to highlight the improvement in inference run-time and energy efficiency of our proposed solution.

• Run-Time Delay Analysis

Table 3 presents the different run-time delays for our FPGA implementation as compared to different GPU implementations. In all cases, the average run-time delay per image $\Delta T_{image}$ is obtained by averaging the total computation time over a large number of input images. It is notable that the FPGA outweighs the GPU in sense of speed for this application in case of a single input image (i.e., Batch size = 1), while the GPU shows less run-time delay for larger batch sizes. An improvement of 4.2 times has been achieved by moving from GPU implementation, in which the average run-time delay per image is 7.01 ms, to the FPGA implementation, in which the average run-time delay per image is decreased to 1.669 ms. For batch sizes greater than 1, the GPU shows smaller run-time delays (hence are faster) as compared with our single input FPGA implementation. However, we will comment on this fact later in the energy efficiency analysis part of the results.

• Energy Efficiency

When it comes to portability, power consumption plays a decisive role. For the sake of comparison, we only consider the dynamic energy consumed for image inference: $E_{image}=\left(P_{total}-P_{idle}\right)*\Delta T_{image}$, where $P_{total}$ is the total power consumed during computation, $P_{idle}$ is the power consumption in idle mode i.e., without inference and $\Delta T_{image}$ is the image run-time. As can be seen in Table 3, the inference using the GPU-based implementation consumes 385.41 mJ as compared to the FPGA-based CNN which consumes only 0.6676 mJ. This implies an improvement of 577.3 times in energy efficiency.

Although the GPU-based implementations achieve faster inference for batch size > 1, the results show that the FPGA is still a valid choice as compared to the GPU for two reasons. First of all, the energy consumption on FPGA is less as compared to all GPU-based implementations shown in Table 3. Secondly, we are not aware of any application, especially real-time, that is tolerant to the delay needed for buffering a big batch of images. In fact, this mode of operation is more suitable for training in batches, which makes GPUs a faster choice for training process. In Table 3, we emphasize on 4 implementations that have special considerations. In addition to the first 2 lines which correspond to batch size = 1, the GPU implementations that correspond to a batch size of 128 and 256 are the most energy efficient and fastest implementations, respectively.

**Figure 9:**
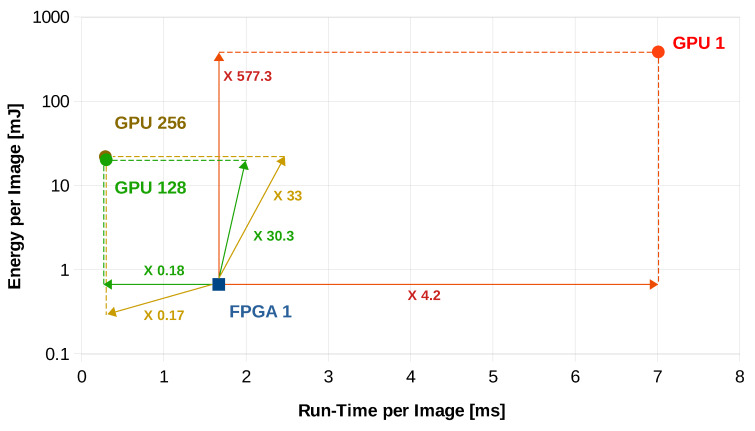
Performance analysis for different batch sizes; FPGA 1 denotes the FPGA implementation performance for batch size 1. Similarly, GPU1, GPU128 and GPU256 denote the GPU implementation performance for batch sizes 1, 128 and 256, respectively. For comparison purposes, we have shown the factors by which the performance differs for different implementations (the numbers on the arrows).

Figure 9 illustrates a visual comparison between FPGA and GPU performance for these 4 important measurements.

#### 6.2.2. Comparison with Other Embedded Platforms

In this part, we provide the reader with a comparison of our method to other embedded platforms. For this purpose, we use the NVIDIA Jetson Xavier which comprises of 512-Core Volta embedded GPU with Tensor Cores, 8-Core ARM v8.2 64-Bit CPU and 16 GB 256-Bit RAM. Furthermore, we deploy the CNN on a Broadcom BCM2837B0, Quad core Cortex-A53 (ARMv8) 64-bit SoC CPU running at 1.4 GHz maximum frequency with 1 GB SDRAM on Raspberry Pi 3B+. In addition to the simple CPU implementation that is expected to be relatively slow, we provide the run-time and energy analysis for the CPU-based inference depending on ONNX-Runtime framework [32]. We run the inference for the testset using different batch sizes (1, 16, 32, 64 and 128), and we measure the average run-time per image as well as the dynamic power and energy needed for this inference.

• Run-Time Delay Analysis

**Figure 10:**
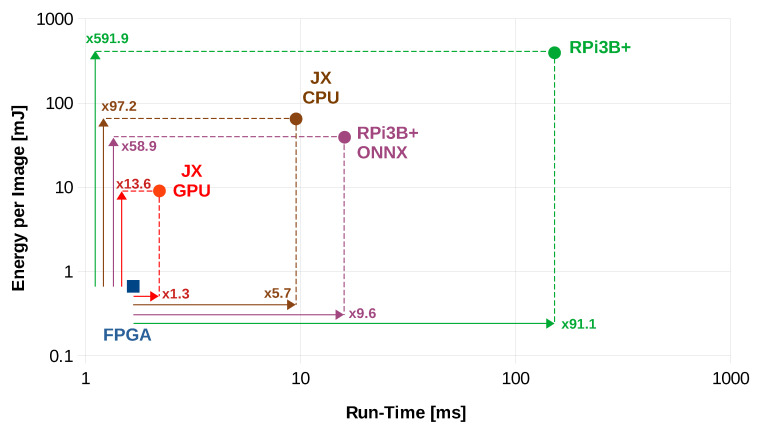
Performance analysis for different embedded platforms; FPGA, JX GPU, JX CPU, RPI3B+ and RPi3B+ ONNX denote the performance of our Xilinx UltraScale+ FPGA, Jetson Xavier embedded GPU, Jetson Xavier embedded CPU, Raspberry Pi 3B+ simple CPU and Raspberry Pi 3B+ ONNX-Runtime-based CPU implementations, respectively. Similarly to Figure 9, the numbers on the arrows illustrate the factors by which the performance differs for different implementations.

It is observable from Table 4 that, for a batch of size 1, the average inference run-time for one image is 2.21 ms as measured on NVIDIA Jetson Xavier’s GPU, while it takes 9.54 ms on the same platform when the CPU is used. Furthermore, it takes 16.04 ms and 151.97 ms when the inference is run on Raspberry Pi 3B+ with and without ONNX-Runtime respectively. This implies that our FPGA implementation is 1.3, 5.7, 9.6 and 91.1 times faster than the Jetson Xavier’s embedded GPU, Jetson Xavier’s embedded CPU, Raspberry Pi 3B+ ONNX-Runtime and Raspberry Pi 3B+ simple embedded CPU implementations, respectively. Figure 10 presents a graphical comparison between our proposed solution and the compared embedded implementations.

**Figure 11:**
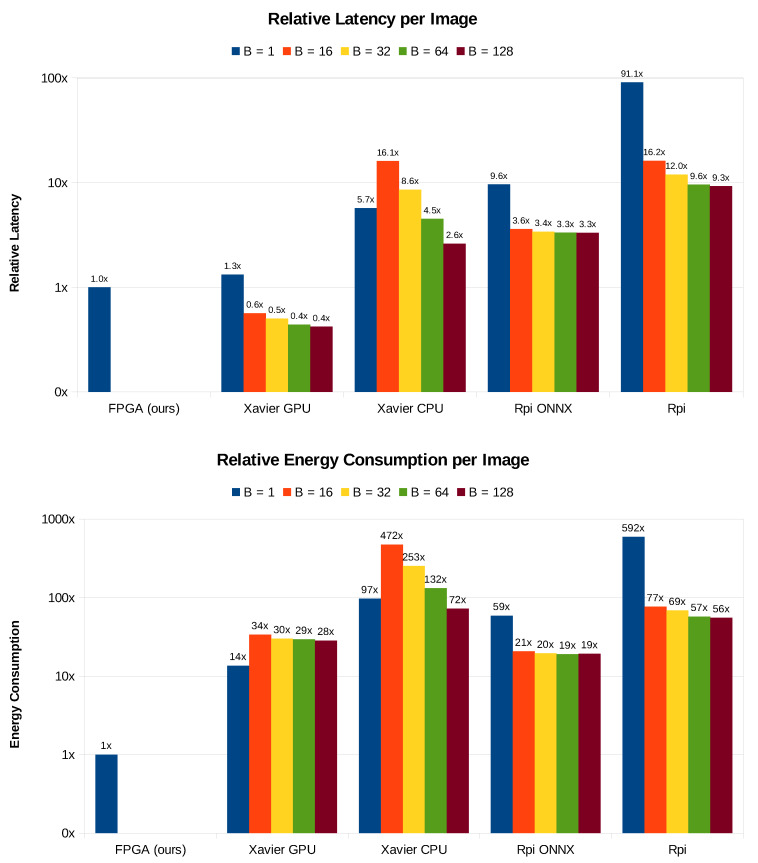
Relative latency and energy consumption per image for different embedded platforms and batch sizes; FPGA, Xavier GPU, Xavier CPU, Rpi and Rpi ONNX stand for our Xilinx UltraScale+ FPGA, Jetson Xavier embedded GPU, Jetson Xavier embedded CPU, Raspberry Pi 3B+ simple CPU and Raspberry Pi 3B+ ONNX-Runtime-based CPU implementations, respectively. The number above each column represents the relative latency or energy consumption with respect to our FPGA implementation.

Instead of plotting the absolute values in Table 4, we take our FPGA implementation latency as a point of reference, and we plot the run-time latency for the different platforms and batch sizes relatively to it in Figure 11. Except for the GPU implementations on Jetson Xavier using batch sizes > 1, which are 1.7× to 2.5× faster, our FPGA implementation outweighs all the other embedded platforms for the given batch sizes. It is notable that the performance of ONNX Runtime on Raspberry Pi 3B+ is nearly the same for the batch sizes 16 to 128. In fact, The inference on Xavier CPU for the batch sizes 16 and 32 (16.1× and 8.6×, respectively) took longer than it does for the batch size 1 (5.7×). The slowest among all these 21 implementations is the Raspberry Pi 3B+ for batch size = 1, which is 91.1× slower than the reference FPGA latency.

• Energy Efficiency and Hardware Resource Analysis

Table 4 shows that our FPGA implementation is more energy efficient when compared with the NVIDIA Jetson Xavier and Raspberry Pi 3B+. Specifically, when the batch size is 1, the average energy demand per image on Jetson Xavier’s GPU and CPU is 9.06 mJ and 64,872 mJ, respectively. Moreover, the average energy consumption for inference on Raspberry Pi 3B+ is 39.30 mJ using ONNX-Runtime while it rises to 395.12 mJ for the simple ARM7 CPU implementation. In other words, our FPGA implementation is 13.6 and 97.2 times more energy efficient than the Jetson Xavier GPU and CPU implementations, respectively. Furthermore, if we compare the Raspberry Pi 3B+ implementation with ours, we observe that the former consumes 58.9 times more energy when running with ONNX-Runtime, while it consumes 591.9 times more energy when running on the CPU without ONNX-Runtime.

For larger batch sizes, Figure 11 shows the energy consumption for each of the aforementioned embedded platforms. The implementation on Jetson Xavier GPU with batch size of 1 was the closest to our FPGA in terms of energy efficiency, although it is 14× less energy efficient. Furthermore, ONNX-Runtime implementation on Raspberry Pi shows almost the same energy consumption when running in batch operation mode. Similarly to the latency behavior, the energy consumption on Jetson Xavier ARM8 is less for batch size of 1 (97×) as compared to the cases of batch sizes 16 (472×) and 32 (253×). Nonetheless, the operation in batch size 64 consumes more energy (132×) when compared to batch size = 1. We observe that the Raspberry Pi ARM7 implementation is the most energy consuming one, requiring 592× more energy than our UltraScale+ implementation. However, it becomes more energy efficient (needs 56× to 77× energy) when running in batch mode.

Overall, it is notable that our FPGA implementation is faster and more energy efficient than the embedded implementations, except for the Jetson Xavier GPU for batch sizes > 1. The area analysis results after Placement and Routing are given in Table 5.

## 7. Discussion

Although the chosen hand pose estimation pipeline in this work is relatively simple, it provides a trade-off between complexity and accuracy and it can be easily extended to a more advanced architecture. Furthermore, this CNN pipeline has been used as a baseline in many works such as DeepPrior [11], DeepPrior++ [33], DeepModel [25] and DeepHPS [34]. In fact, other hand pose estimation algorithms with higher joint regression accuracy can be found in the literature. There exist a number of techniques that are expected to enhance the estimation accuracy such as dilation as well skip and merge connections. We did not use these techniques in our hardware implementation as we mainly focused on the hardware optimization of an already existing algorithm. However, we preferred to enrich the reader with an overview of these two techniques and the related architectural changes.

### 7.1. Dilation

In dilated convolution, the convolutional kernel is expanded by inserting fixed gaps between its weights horizontally and vertically. This differs from the standard convolution where the convolutional kernel is convoluted with a region of adjacent input values. In the special case of the standard convolution, where dilation is equal to 1, the height and width of the line buffer are given as the kernel height ($K_{H}$) and the input width ($W_{in}$) respectively. However, in general case (i.e., for dilation d≥1), the height of the line buffer becomes $K_{H}+\left(K_{H}-1\right)\times \left(d-1\right)$. Despite this change, the size of the window buffer does not have to be tampered with, since the amount of input values that are needed to be convoluted depend only on the kernel size. However, the difference lies in the sampling logic that picks the desired values from the line buffer to the window buffer. The effect of the dilation on the layer’s idle time can be obtained by differentiating $\Delta_{idle}$ in Equation (6) with respect to the dilation *d*:

(10) $$\begin{array}{cc} \frac{\partial \Delta_{idle}}{\partial d} & =-2\frac{{\left(K_{H}-1\right)}^{2}}{s^{2}}d+2\frac{\left(H_{in}-1\right)\left(K_{H}-1\right)}{s^{2}} \end{array}$$

The quantity $\partial \Delta_{idle}/\partial d$ is positive for all $d<\left(H_{in}-1\right)/\left(K_{H}-1\right)$. In practical cases, where d≤3 and $H_{in}$ is a few multiples of $K_{H}$, the dilation increases the idle time in this particular layer.

### 7.2. Skip and Merge Connections

Since the hardware architecture in this work is mainly based on the streaming paradigm, skip connections (as in ResNet [35]) and merge connections (as in DenseNet [36]) should be implemented on stream level. Therefore, we introduce two fake layers, namely the Branching layer and the Merging layer. For the skip connection, the branching layer should be inserted at the point where the stream is split into two or more branches. This layer is mainly responsible for repeating each received input value a number of times equal to the number of branches. In this case, each instance value is streamed out from the branching layer in a particular branch. On the other hand, the merging layer is needed to concatenate the different branches into a unified branch. This layer reads a single value from each input branch and then, based on the desired functionality, can sum the inputs or multiplex them consecutively in the output stream. In fact, each branch might have a different computation complexity, and therefore, it might suffer from a different propagation delay. This can result in merging a particular value from an incoming branch with a prior or posterior value from another branch in the merging layer. In other words, the streaming order among all branches must be maintained and the synchronization among the different branches is required. One way to achieve that would be to perform a delay analysis for each branch and consequently insert buffers of certain lengths in the faster branches. For further details, we advise the reader to check the work by Chidananda et al. [37].

## 8. Conclusions

Real-time, energy efficient 3D hand pose estimation is becoming important in many research fields, especially human machine interaction. Many researches have proposed various software implementations for hand pose estimation algorithms based on high-performance GPUs. However, software environments suffer from several limitations such as run-time latency, power consumption and difficulty of portability. In this work, we have taken CNN-based hand pose estimation into another level by providing an efficient hardware implementation for it. The first step in this work was to provide a trained software model of the CNN using PyTorch. This model is used as a reference in later steps. Then, the size of this model was reduced by scaling down the number of kernels and effectively quantizing the network model parameters, which resulted in a significantly compressed model for a negligible decrease in accuracy. Afterwards, we provided an optimized hardware streaming architecture for the CNN which was then implemented on Xilinx UltraScale+ MPSoC FPGA. Our results have shown that the FPGA achieves better performance than its software counterpart, which run on high-performance GPU for the same batch size. In terms of inference time and power consumption, we have illustrated that FPGA is a suitable platform for portable Hand Pose Estimators, since this implementation is 4.2× faster, 5.3× smaller and 577.3× more energy efficient as compared to the GPU for the same batch size for a slight degradation of 3% in accuracy.

## 9. Future Work

From software point of view, other CNN topologies can be experimented for the same application using the same implementation methodology. The usage of deeper CNNs with more cascaded layers might reduce the lower limits of quantization bitwidths resulting in shorter bitwords and eventually a smaller network model size with higher performance. This might even allow the design to go to the extra mile in design space by binarizing the model instead of just quantizing it. Presumably, other quantization methods might be able to further reduce the size of the same CNN used in this work. A small delay appears as a bottleneck when the input image or a feature map is streamed to a layer in raster scan (i.e., line per line) order. Moreover, it is an open question to try other scanning methods and decide which method is more effective in terms of time and space. Finally, we aim to further improve the accuracy of hand pose estimation by switching to a deep network architecture that comprises skip connections, similarly to ResNet, and concatenations, similarly to DenseNet. This architecture is still pending in the evaluation test phase and we are planning to publish it in an upcoming article.

## Figures and Tables

**Table 1:** Hyper parameters for training a full-precision CNN.

Hyper Parameters	Value
No. Kernels per Conv Layer	8
No. Epochs	500
Optimizer	SGD
Loss Function Criterion	MSE
Batch Size	256
Learning Rate	0.005
Momentum	0.9

**Table 2:** Quantized vs. full-precision CNN volume.

Layer	Total No. Parameters	Full-Precision Size	Quantized Size
Convolutional 1	208	6.5 Kb	2.4 Kb
Pooling 1	-	-	-
Convolutional 2	208	6.5 Kb	2.4 Kb
Pooling 2	-	-	-
Convolutional 3	80	2.5 Kb	0.9 Kb
Fully Connected 1	1,180,672	36 Mb	6.8 Mb
Fully Connected 2	1,049,600	32 Mb	6.0 Mb
Fully Connected 3	95,325	2.9 Mb	559 Kb
**Total**	**2,326,093**	**71 Mb**	**13.3 Mb**

**Table 3:** FPGA vs. GPU batch performance analysis.

Platform	Batch Size	Run-Time [ms]	Energy [mJ]
FPGA (ours)	**1**	**1.669**	**0.6676**
GPU	**1**	**7.01**	**385.41**
	32	0.53	30.55
	64	0.35	21.86
	**128**	**0.30**	**20.20**
	**256**	**0.29**	**21.99**
	512	0.30	22.10
	1024	0.31	21.80
	2048	0.30	35.80
	4096	0.30	35.86
	8192	0.30	40.49

**Table 4:** FPGA vs. different embedded platforms performance analysis.

Platform	Batch Size	Run-Time (ms)	Energy (mJ)
FPGA (ours)	1	1.669	0.6676
NVIDIA Jetson Xavier (GPU)	1	2.21	9.06
	16	0.94	22.51
	32	0.84	20.05
	64	0.73	19.64
	128	0.70	18.94
NVIDIA Jetson Xavier (CPU)	1	9.54	64,87
	16	26.84	315.36
	32	14.28	169.20
	64	7.51	88.27
	128	4.36	48.33
RaspberryPi 3B+ (ONNX-Runtime)	1	16.04	39.30
	16	6.01	13.82
	32	5.66	13.03
	64	5.56	12.67
	128	5.53	12.84
RaspberryPi 3B+	1	151.97	395.12
	16	27.00	51.30
	32	19.95	45.89
	64	15.98	38.36
	128	15.45	37.07

**Table 5:** Resource utilization summary on XCZU9EG.

Resources	Used	Available	Util
CLB LUTs	21,432	274,080	8%
CLB Flip Flop	15,104	548,160	3%
Block RAM Tile	772.5	912	85%
DSPs	20	2520	1%

**Figure A1:**
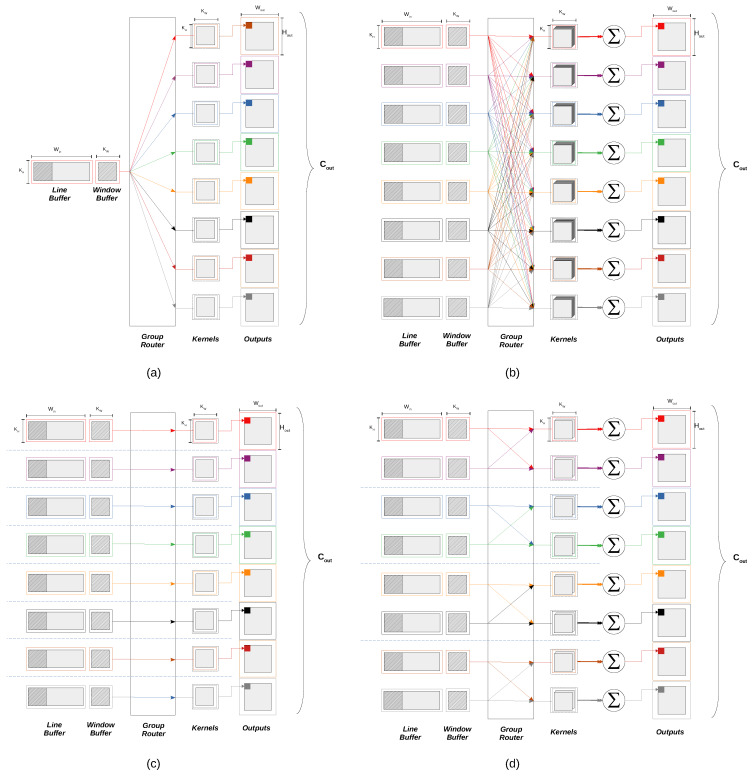
Group Convolution for 8 output feature maps. This figure shows 4 different examples to obtain 8 output feature maps. (**a**) The 2D convolution for a single input feature map. In this case, the input consists of a single group and 8 kernels. Each kernel is convoluted with the input feature map to produce an output feature map. (**b**) The case of 8 input feature maps and a single input group, where 64 kernels are split into 8 groups and each group is convoluted with the input. The summation is necessary to obtain the desired number of output feature maps. (**c**) Depthwise convolution. The input is split into 8 groups and each output feature map is computed depending form a single input feature map. (**d**) Another example of group convolution in which the input is split into 4 groups. In this case, a total number of 16 kernels are required and each input group is convoluted with a set of 4 kernels. We ignored the summation in (**a**,**c**) since the result of each convolution is a single output feature map.

## Appendix A

### Appendix A.1. Stride Algorithm

Algorithm A1 presents a code snippet for stride operation. The row and column counters (*i* and *j*) track the location of the input value within the input feature map. The operation is allowed to be performed only when the row and column counters are multiples of the vertical and horizontal strides respectively. The row and column counters are initialized to the negative values of the kernel dimensions. This prevents the operation from being executed before the data is available in the line buffer.

**Algorithm A1:** Stride-Aware Convolution and Max-Pooling

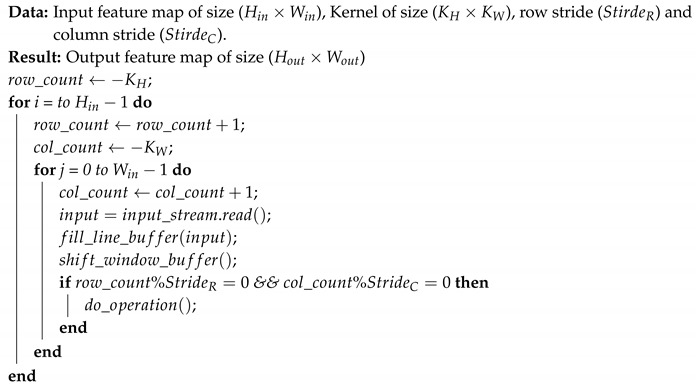

### Appendix A.2. Zero Padding Algorithm

Algorithm A2 presents a code snippet for zero padding. The padding decision is based on the values of the row and column counters (*i* and *j*). These counters track the horizontal and vertical locations of each input value relatively to the corresponding output feature map. That is why the limits of the counters are the dimensions of the output feature map instead of the input feature map.

**Algorithm A2:** Zero Padding

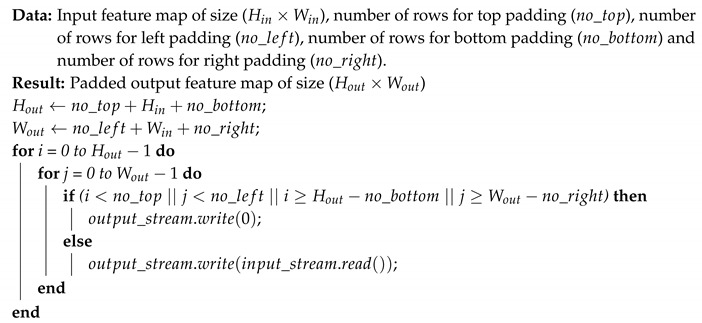

### Appendix A.3. Group Convolution

In case of the first convolutional layer, which produces 8 output channels from a single input channel in our work, 8 convolutional kernels are used for the single input group (Figure A1a). While in the second and third convolutional layers, the standard convolution requires 64 kernels to produce 8 output channels (Figure A1b). As mentioned in Section 3.1, depthwise convolution is used to reduce the needed storage area as well the computation complexity while performing the convolution. Specifically, the 8 input channels are grouped into 8 groups, each containing one single feature map. The number of kernels is reduced from 64 to 8 in this case, where each kernel is convoluted with a single input feature map (Figure A1c). In all cases, the number of kernels needed to perform the convolution is $C_{out}\times C_{in}/G$, where $C_{out}$ is the number of output channels, $C_{in}$ is the number of input channels and *G* is the number of input groups. The group convolution has no effect on the idle time $\Delta_{idle}$ of the layer since it does not effect the buffering scheme in the line buffer.
